# Neuroendocrine tumor presenting like lymphoma: a case report

**DOI:** 10.1186/1752-1947-5-506

**Published:** 2011-10-05

**Authors:** Riccardo Del Vescovo, Roberto Luigi Cazzato, Sofia Battisti, Francesco D'Agostino, Bruno Vincenzi, Rosario Francesco Grasso, Bruno Beomonte Zobel

**Affiliations:** 1 Department of Radiology, Campus Bio-Medico, University of Rome, Italy; 2 Department of Oncology, Campus Bio-Medico, University of Rome, Italy

## Abstract

**Introduction:**

Neuroendocrine tumors are a rare but diverse group of malignancies that arise in a wide range of organ systems, including the mediastinum. Differential diagnosis includes other masses arising in the middle mediastinum such as lymphoma, pericardial, bronchogenic and enteric cysts, metastatic tumors, xanthogranuloma, systemic granuloma, diaphragmatic hernia, meningocele and paravertebral abscess.

**Case presentation:**

We present a case of 42-year-old Caucasian man with a neuroendocrine tumor of the middle-posterior mediastinum and liver metastases, which resembled a lymphoma on magnetic resonance imaging.

**Conclusion:**

The differential diagnosis in patients with mediastinal masses and liver lesions should include neuroendocrine tumor.

## Introduction

Neuroendocrine tumors (NETs) are a rare but diverse group of malignancies that arise in a wide range of organ systems. This can therefore include the mediastinum [1,2].

Differential diagnosis includes other masses arising in the middle and posterior mediastinum, such as lymphoma, pericardial, bronchogenic and enteric cysts, metastatic tumors, xanthogranuloma, systemic granuloma, diaphragmatic hernia, meningocele and paravertebral abscess. When a large mass is discovered, pathological conditions of the anterior mediastinum, such as thymoma, germ cell tumors, intrathoracic goiter, lipoma, lymphangioma and aortic aneurysm, should also be considered [3,4]. We present a case of a NET of the middle-posterior mediastinum with liver metastases, which resembled a lymphoma on magnetic resonance imaging (MRI).

## Case presentation

**Figure 1:**
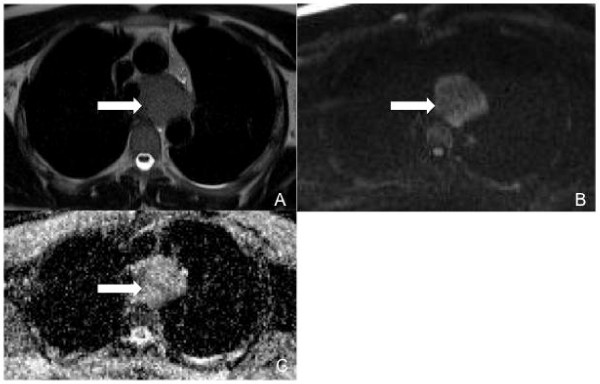
**MRI of the patient's mediastinum**. (A) T2-weighted half Fourier acquisition dingle shot turbo spin echo (HASTE) sequence on axial plane shows a mediastinal solid mass. (B,C) DWI at b1000 shows a homogeneous high signal intensity and a non-homogeneous low signal intensity on ADC map (white arrows).

**Figure 2:**
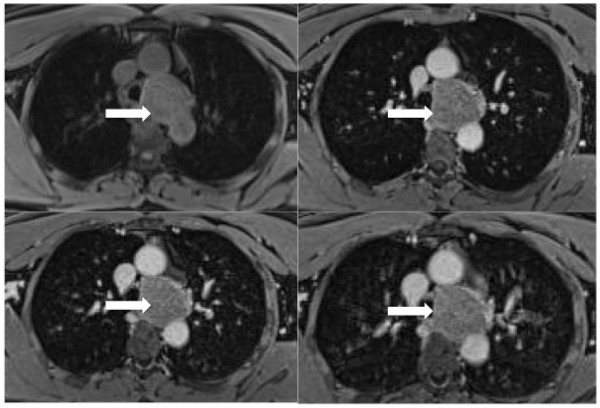
**T1-weighted volumetric interpolated breath-hold examination (VIBE) sequences on axial planes**. The post-contrast T1-weighted images show poor enhancement within the solid lesion (white arrows).

**Figure 3:**
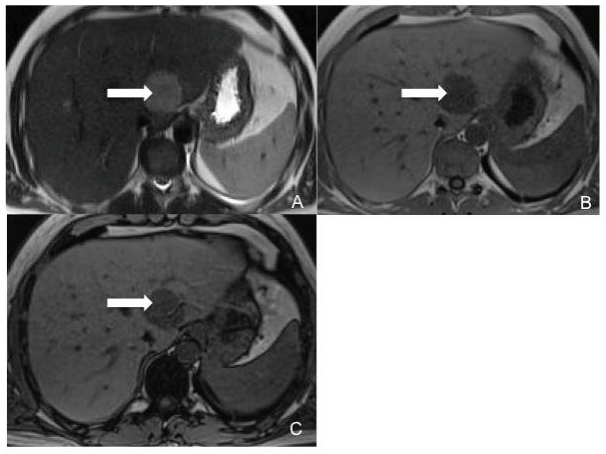
**T2-weighted HASTE sequence on axial plane**. Shows one of the solid liver lesions in the II segment, with the same patterns of signal intensity showed by the mediastinal mass on the (A) T2-weighted and (B,C) T1-weighted images before contrast median injection (white arrows).

**Figure 4:**
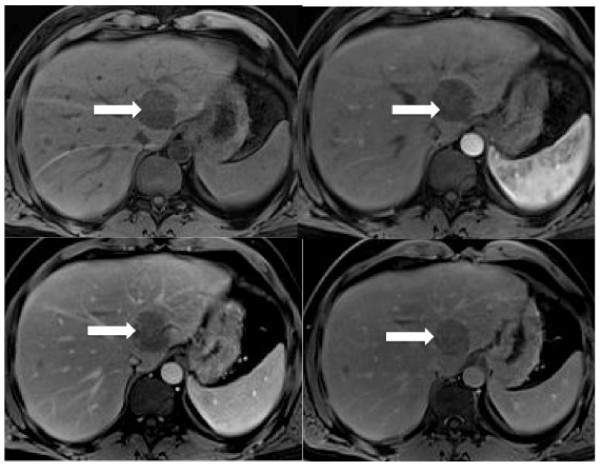
**T1-weighted VIBE sequences obtained before and after contrast median injection showed a poor enhancement within the solid lesions (white arrows)**.

A 42-year-old Caucasian man was referred to our institution because of a recent onset of dysphonia. He had no significant past medical history and his physical examination was unremarkable. His erythrocyte sedimentation rate was increased (38 mm/h; normal range 0.00 to 17.00 mm/h). His blood cell count was normal as were the results of his other laboratory tests, including human immunodeficiency virus serology, urea and electrolytes, liver function tests and lactate dehydrogenase levels. Chorionic gonadotropin, neuron-specific enolase (NSE), prostate specific antigen, carcinoembryonic antigen, cancer antigen-125 and cancer antigen-19.9 were also tested and found to be negative. A noncontrast and postcontrast MRI of his mediastinum revealed a 6 cm × 4 cm × 5 cm solid lesion in the left paratracheal area, which appeared to enter and widen the aortopulmonary space. It also displaced his distal trachea and branches of his left bronchus medially and inferiorly, respectively. On T2-weighted images, the mass showed homogeneous high signal intensity. In contrast, a low intensity signal was noted on T1-weighted images and on the apparent diffusion coefficient (ADC) map when diffusion weighted images (DWI) were considered (Figure 1). The postcontrast T1-weighted images showed poor enhancement within the solid lesion (Figure 2). No lymph node involvement was noted in other areas of his chest. The MRI was then extended to his abdomen and showed some solid lesions in the II, III and VIII hepatic segments. These had the same patterns of signal intensity observed in the mediastinal mass on the T1- and T2-weighted images, before and after administration of contrast medium (Figures 3 and 4). No vascular or biliary structures were invaded by the hepatic mass and no abdominal lymph node involvement was noted.

According to the radiological features, the diagnosis was suspected lymphoma. Therefore, bioptical sampling of the hepatic lesion was performed (Precisa: 18G × 2 cm). Microscopic examination revealed small sized cells, some of them spindle-shaped, which were positive for cytokeratins AE1/AE3, chromogranin, NSE and synaptophysin on immunohistochemical analysis. Ten percent of the cells expressed Ki67 and none of them showed a CD3-CD20 pattern. According to the pathological response, the diagnosis was a NET with hepatic metastasis.

Systemic chemotherapy based on cisplatin plus etoposide was started and then replaced by a new scheme based on capecitabine because of the progression of the disease, which was particularly noticeable in his liver. Therefore, a transarterial chemoembolization was considered as a suitable option to control the hepatic disease and then performed by emulsifying polyvinyl alcohol particles and doxorubicin [5].

## Discussion

NETs are a rare but diverse group of malignancies that arise in a wide range of organ systems. Gut-derived NETs arise from the diffuse endocrine system and, according to the 2002 World Health Organization classification system [6], are most simply classified based on their site of origin as either 'functional' (symptomatic hormone secretion) or 'non-functional' (no symptomatic hormones). Many investigators still find it practical to use the categorization of NETs based on the embryologic origin into foregut tumors (bronchi, stomach, pancreas, gallbladder, duodenum), midgut tumors (jejunum, ileum, appendix, right colon), and hindgut tumors (left colon, rectum). Although there has been continued improvement in detection, NETs (most commonly, midgut NETs) have frequently metastasized to the liver by the time of diagnosis. A diverse range of imaging modalities can be used for the assessment of NETs.

The case presented here was a suspected malignant mediastinal tumor, because of the young age of our patient and the presence of dysphonia. For this reason, MRI was also extended to his abdomen. Radiological features of low vascularization and the absence of involvement of the vascular and biliary structures shown by the hepatic and mediastinal lesions led to a first diagnostic hypothesis of mediastinal lymphoma with hepatic spread. The latter hypothesis was also supported by the fact that more than 80% of patients with Hodgkin's disease and almost 45% of patients with non-Hodgkin's lymphoma show intrathoracic involvement [7,8]. Moreover, in the thorax, lymphoma frequently involves the anterior mediastinal and paratracheal regions [9]. However, due to the absence of systemic symptoms and the presence of extrathoracic involvement at presentation, which usually occurs in immunocompromised patients, bioptical sampling of the hepatic lesions was performed and revealed the presence of a neuroendocrine malignancy.

A multimodality approach is optimal for detecting the primary tumor and metastases and can include multidetector computed tomography (CT), functional imaging, MRI, ultrasonography, endoscopy, digital subtraction angiography and venous sampling.

For the evaluation of liver metastases, the use of triple-phase multidetector CT and contrast material-enhanced MRI is suggested to establish a baseline, which can help assess disease extent and allow post-treatment comparison. The arterial anatomy of the liver and portal vein patency can also be determined. Liver metastases have low signal intensity on T1-weighted MRI and high signal intensity on T2-weighted images [10].

The treatment of patients with liver metastases is complex, and there are often several factors that must be taken into account. Curative surgery should always be considered but is rarely possible due to the diffuse nature of the disease.

NETs are relatively slow-growing tumors; therefore, patients can survive for several years with current treatment strategies. Interventional radiology has played an increasingly important role.

Interventional radiologists could approach liver metastases by performing procedures such as embolization, chemoembolization, targeted radionuclide therapy and local ablative techniques (radiofrequency ablation, cryotherapy and percutaneous ethanol injection) [11]. Radiological treatments may be combined with curative surgery, which should always be considered as a treatment option. Chemoembolization should not be performed in patients in whom portal vein obstruction, liver insufficiency, abscess, previous biliary anastomoses and biliary obstruction have been discovered during the diagnostic phase. Whether chemoembolization should be used rather than embolization is still a matter of debate, since recent evidence has emerged that the addition of intraarterial chemotherapy to embolization does not improve the outcome in patients with carcinoid tumors [12]. Radionuclide therapy could be proposed if other treatments are not possible or have failed; positive uptake of ^111^In-pentetreotide has been proven on a previous scintigraphy and trained staff is available. Radionuclide therapy should not be performed in cases of pregnancy and lactation, or bone marrow dysfunction (hemoglobin below 10 g/dL, white blood cell count fewer than 2 × 10^3^/mL, platelet count below 100/mL) [13]. Ablative therapies should be performed as adjuncts to surgical resection, for treatment of patients with unresectable tumor, palliation and recurrence after surgical resection or prior ablation [14,15].

## Conclusion

Mediastinal neuroendocrine tumors are a challenging disease for clinicians and radiologists. Although the clinical approach is still the mainstay both in the diagnostic and therapeutic phases, the radiological approach could provide valuable support. The differential diagnosis in patients with mediastinal masses and liver lesions should include neuroendocrine tumor.

## Consent

Written informed consent was obtained from the patient for publication of this case report and any accompanying images. A copy of the written consent is available for review by the Editor-in-Chief of this journal.

## Competing interests

The authors declare that they have no competing interests.

## Authors' contributions

BV, FDG and SB analyzed and interpreted the patient data. RDV, RFG and BBZ performed the thorax and abdominal magnetic resonance. RDV and GC were major contributors in writing the manuscript. All authors read and approved the final manuscript.
